# The HUPO Proteomics Standards Initiative Meeting: Towards Common Standards for Exchanging Proteomics Data

**DOI:** 10.1002/cfg.232

**Published:** 2003-02

**Authors:** Sandra Orchard, Paul Kersey, Henning Hermjakob, Rolf Apweiler

**Affiliations:** EMBL Outstation–European Bioinformatics Institute Wellcome Trust Genome Campus Hinxton Cambridge UK

## Abstract

The Proteomics Standards Initiative (PSI) aims to define community standards for data representation in proteomics and to facilitate data comparison, exchange and verification. Initially the fields of protein–protein interactions (PPI) and mass spectroscopy have been targeted and the inaugural meeting of the PSI addressed the questions of data storage and exchange in both of these areas. The PPI group rapidly reached consensus as to the minimum requirements for a data exchange model; an XML draft is now being produced. The mass spectroscopy group have achieved major advances in the definition of a required data model and working groups are currently taking these discussions further. A further meeting is planned in January 2003 to advance both these projects.


**Comparative and Functional Genomics**
*Comp Funct Genom* 2003; **4**: 16–19.
Published online in Wiley InterScience (www.interscience.wiley.com). **DOI:** 10.1002/cfg.232

**Feature**

### Meeting Review: The HUPO Proteomics Standards Initiative meeting: towards common standards for exchanging proteomics data

Hinxton, Cambridge, UK, 19–20 October 2002

Sandra Orchard, Paul Kersey, Henning Hermjakob* and Rolf Apweiler
*EMBL Outstation–European Bioinformatics Institute, Wellcome Trust Genome Campus, Hinxton, Cambridge, UK*

**Correspondence to:
Henning Hermjakob, EMBL Outstation–European Bioinformatics Institute, Wellcome Trust Genome Campus, Hinxton, Cambridge, UK.
E-mail: Henning.Hermjakob@ebi.ac.uk*

Received: 14 November 2002
Accepted: 14 November 2002

### Abstract

**The Proteomics Standards Initiative (PSI) aims to define community standards for data representation in proteomics and to facilitate data comparison, exchange and verification. Initially the fields of protein–protein interactions (PPI) and mass spectroscopy have been targeted and the inaugural meeting of the PSI addressed the questions of data storage and exchange in both of these areas. The PPI group rapidly reached consensus as to the minimum requirements for a data exchange model; an XML draft is now being produced. The mass spectroscopy group have achieved major advances in the definition of a required data model and working groups are currently taking these discussions further. A further meeting is planned in January 2003 to advance both these projects. Copyright © 2003 John Wiley & Sons, Ltd.**

**Keywords:** **proteomics; spectroscopy; protein–protein interactions**

### Introduction

The Proteomics Standards Initiative was established following a meeting in April 2002, jointly organized by HUPO and NAS, at which the urgent need for standardization of proteomics data was recognized. **Rolf Apweiler (Sequence Database Group, European Bioinformatics Institute)** opened the proceedings by explaining that a decision had been made to address these issues initially in the fields of mass spectroscopy and protein–protein interactions (PPI). This inaugural meeting of the Proteomics Standards Initiative brought together representatives from the database producer, user and software producer communities, who were seen as essential in establishing and maintaining the required standards and who were jointly charged over the 2 days of the meeting with laying the groundwork that would enable these objectives to be met.

The delegates listened to a short presentation by **Alvis Brazma (EBI)**, outlining the successful standardization of microarray data in the MGED process, before splitting into two working parties to address the issues facing their respective fields.

### Protein–protein interactions (PPI) group

The session commenced with a brief introduction from each of the PPI databases represented at the meeting as to the ethos and coverage of their particular product. This included presentations by representatives from Hybrigenics SA, DIP, BIND, MINT, GIN-DB, PPID and IntAct, a public repository of PPI data that will be launched by the EBI early in 2003. The meeting was then thrown open to address a number of key issues.

### Is there a requirement for a community standard?

Data exchange is essential for the purposes of data comparison, benchmarking and quality control, all of which are particularly important in a field like protein–protein interaction, where the

Copyright © 2003 John Wiley & Sons, Ltd.

standard high-throughput methods are known to yield high false-positive and false-negative rates. A community standard should allow simple access to core protein interaction data, while being extensible to exchange data with a high level of detail. Many users will require only simple indexing and interface systems; larger organizations will have requirements that are more complex but will have the infrastructure to develop much of this themselves. The confidentiality of data could be seen as an issue that might inhibit organizations from contributing; however, this question has already been addressed by the various sequence databases where entries can be flagged and retained by the parent database until permission is given for release. It was recognized early on in the discussions that a minimum standard for data exchange needed to be developed and a formal mechanism for monitoring and maintaining this standard put in place. Valuable lessons can be learned in this area from MGED's experience of defining a minimal standard for the exchange of microarray data.

### Definition of use cases

The potential use of the data has to be understood before the minimum common standard can be defined. Most of the groups represented at the meeting were interested in making graphical representations of PPIs and in making interspecies comparisons based on sequence or structural homology. To compare data from different systems, a correct description of the source systems is essential, including details of species, strain and, in some cases, tissue, cell type and disease state. Domain identification and the dynamic properties of PPIs were also common requirements, whilst the functional outcome of PPIs and the effects of sequence variations and posttranslational modifications were seen as desirables. Some users have a requirement for in-depth experimental detail; however, this was felt to be beyond the scope of a data exchange format and would have to be retrieved from the literature. Links to public databases were seen as essential when available but would not be made mandatory, since this would compromise the transfer of unpublished data between collaborating laboratories.

### Outline data structure

The need for a multi-level approach was soon recognized, with Level 1 designed to fulfil basic requirements and be suitable for rapid implementation, whilst subsequent levels will contain more features, yet remain compatible backwards. The interchange format will need to be able to represent both binary and $n$-ary (complex) interactions. The topology of the latter would then be described within each set.

Each Interchange Format Record will report one or more interactions supported by one or more experiments. Predicted interactions are allowed and will be clearly flagged. Wherever the sequence of the interactors is available in public databases, appropriate cross-references should be given. The sequence should be given in the interaction record when it is not available from public databases, and may always be given.

Each entry will need to contain the accession number of its parent database. Parent databases will be identified by a prefix. This will require a registry service, which will have to be recognized and maintained. It is proposed to use PSI/HUPO as the authority for this and a host site will have to be established, which can be accessed by databases wishing to submit data.

The standardization of experimental design provides a particularly complex set of issues for the field of PPI, in which researchers use a host of diverse techniques and practices. Level 1 of the standard will not attempt to provide a full description of the experimental design, but will provide the means to clearly classify the experiments through hierarchical controlled vocabularies.

A work group has been set up to develop common controlled vocabularies for experimental methods and other attributes of protein interaction data. These will be used by the interaction data standard and will be made available via the Global Open Biological Ontologies (GOBO) website.

To capture a larger part of the interaction data that is generated worldwide, the support of major biochemistry and proteomics journals in this process is seen as crucial. It is proposed that, once a PSI PPI level 1 standard has been established, the major public database providers will collectively approach journals and funding agencies to request that deposition of published interaction data in public databases will be strongly encouraged as

Copyright © 2003 John Wiley & Sons, Ltd.

part of the publication process. This would be similar to the deposition requirement for nucleotide sequence data, and the current encouragement to deposit DNA microarray data.

### PPI molecular interaction interchange format record structure

The structure of an Interchange Format record defining both mandatory and optional fields was discussed in great detail and a draft document was produced. A small working party was formed to produce an XML draft of this consensus, which will then be further refined and finally presented to members of the PSI at a meeting in January. The PPI group aim to have a publicly available version of the level 1 format available by Spring 2003.

### Mass spectrometry

This session discussed two questions — the use of standards in the field of mass spectrometry and the potential use of a public data repository for mass spectrometry data.

Following presentations on various aspects of mass spectrometry by **Alexey Nesvizhskii (ISB, Seattle, MI)**, **Arkadiusz Nawrocki (CPA, Odense, Denmark)** and **Rulin Zhang (SynX Pharma, Toronto, Canada)**, the group received a demonstration of PEDRo, a tool developed at the University of Manchester to capture data and metadata from proteomics experiments that include mass spectrometry as one component. PEDRo has been designed according to the MGED guidelines and has a similar scope to the microarray data model, capturing the complete process of scientific experiment from hypothesis formation through to peak identification. A consideration of PEDRo led to the discussion as to whether a single repository would encompass the diverse needs of mass spectrometry in the context of proteomics or whether separate standards for each type of experiment, with separate repositories for each type of data, would be required. As the issues became apparent, questions of feasibility were also raised. Examples were given of ambitious plans to design software that supported data from all types of proteomics experiments, which had eventually been replaced by projects aimed at capturing only one particular workflow.

Mass spectrometry data exists at many levels, from raw data, through peak lists and peptide identification, to protein identification; on top of this is the desire to mine data. Huge amounts of variation (and manual interpretation) exist in the processes that effect these transformations. The following specific points were discussed in more detail.

### The purpose of new repositories

One projected use was to provide an audit trail for publications, so that the producers of bulk or complex data would be able to fully describe (and be held to account for) methodologies that could not appear in print medium; this would require the cooperation of journals. Another purpose could be to allow the user to explore/mine the data, preferably in a biological context. Important concepts here are 'the minimal description of the experiment' and 'validation criteria'.

### How many repositories?

A component-based approach, with different repositories for different types of proteomics experiment, was considered, but fears were expressed that this would disrupt the audit trail, or make biological interpretation of the data impossible. How to go about capturing the meaningful results of an experiment that resulted in the conclusion that two proteins interact, without a wasteful overlap with PPI databases, was discussed at intervals throughout the meeting.

### Would the users enter all the data?

The hope was expressed that if a standard could be produced, LIMS systems might automatically produce compliant output. However, proteomics is often not fully automated and many data points might be missing.

### Participation of equipment manufacturers and other parties

The view was expressed that the participation of equipment manufacturers was essential to the ultimate success of any new standard. In areas such as hypothesis description and preliminary sample preparation, substantial opportunities for overlap

Copyright © 2003 John Wiley & Sons, Ltd.

with other groups involved in standardization were perceived, and enthusiasm expressed for taking these forward.

### Error rates

There is little public awareness among potential users of the data of problems, such as estimating error rates and the statistical complexity in producing the final protein identifications. A need to raise community awareness of these issues was recognized.

Three work groups have now been established:

- *Group 1* will work on the definition of mass spectrometry data, and the subsequent data analysis, as far as protein identification. A draft model has been produced, which includes the facility for recursive analysis and refinement of the peak list.
- *Group 2* are modelling the process of sample preparation, considering the overall workflow of proteomics experiments in which 'mass spectrometry' was one component, up to the point where a sample is ready to be loaded into the spectrometer. Again, a recursive model has been used, whereby a sample could undergo many cycles of preparative steps.
- *Group 3* are considering likely user demands of any implemented system. The interests of both expert mass spectrometrists and biological users are being considered. A system should support the ability to query with peak lists, and with known sample compositions, against the results of previous experiments; and should also allow users to query across experiments to observe the concomitant changes in identified species.

The findings of these working groups will be presented during the HUPO conference in November 2002 and the way forward can then be discussed with input from the wider proteomics community, who will be attending that meeting.

### Conclusions

There was a remarkable consensus between delegates attending the PSI meeting to the effect that valuable data would be lost without public repositories and common interchange formats making information accessible to the scientific community. Major progress was made in the field of protein–protein interactions, with a draft exchange format being produced and work on an XML version in progress. The mass spectroscopy group has to undertake more groundwork, to establish common needs and requirements, to identify what data is appropriate for public access and the degree of supplementary information which is required to be stored alongside, but important advances have been made and it is hoped that this group will have preliminary results by early 2003.

All such efforts require support from the user community and from the scientific press and funding agencies. Members of the PSI will be actively canvassing such collaboration, but input is welcome from any quarter. Anyone wishing to become involved is invited to visit **http://psidev.sf.net**, to participate in the discussion groups listed, and to contribute to the further development of community standards for proteomics data. A further meeting of the PSI is planned for 22–24 January 2003 in Hinxton, Cambridge, UK. Details will be published via the website.

### Related websites

BIND: **http://bind.ca/**
DIP: **http://dip.doe-mbi.ucla.edu/**
Hybrigenics: **http://www.hybrigenics.fr**
IntAct Project: **http://www.ebi.ac.uk/intact/**
MINT: **http://cbm.bio.uniroma2.it/mint/**
MGED: **http://www.mged.org**
PPID: **http://www.anc.ed.ac.uk/mscs/PPID**
PSI: **http://psidev.sf.net/**

---

The Meeting Reviews of *Comparative and Functional Genomics* aim to present a commentary on the topical issues in genomics studies presented at a conference. The Meeting Reviews are invited; they represent personal critical analyses of the current reports and aim at providing implications for future genomics studies.

---

Copyright © 2003 John Wiley & Sons, Ltd. 

